# Selective Histone Deacetylase 6 Inhibitor 23BB Alleviated Rhabdomyolysis-Induced Acute Kidney Injury by Regulating Endoplasmic Reticulum Stress and Apoptosis

**DOI:** 10.3389/fphar.2018.00274

**Published:** 2018-03-26

**Authors:** Yuying Feng, Rongshuang Huang, Fan Guo, Yan Liang, Jin Xiang, Song Lei, Min Shi, Lingzhi Li, Jing Liu, Yanhuan Feng, Liang Ma, Ping Fu

**Affiliations:** ^1^Division of Nephrology, Kidney Research Institute, West China Hospital of Sichuan University, Chengdu, China; ^2^Core Facility of West China Hospital, West China Hospital of Sichuan University, Chengdu, China; ^3^Laboratory of Clinical Pharmacology, West China Hospital of Sichuan University, Chengdu, China; ^4^Department of Pathology, West China Hospital of Sichuan University, Chengdu, China

**Keywords:** rhabdomyolysis, acute kidney injury, histone deacetylase 6 inhibitor, endoplasmic reticulum stress, apoptosis

## Abstract

Histone deacetylase 6 (HDAC6) contributed to the pathogenesis of rhabdomyolysis-induced acute kidney injury (AKI) and selective inhibition of HDAC6 activity may be a promising strategy for the treatment of AKI. Compound 23BB as a highly selective HDAC6 inhibitor was designed, synthesized by our lab and exhibited therapeutic potential in various cancer models with good safety. However, it remained unknown whether 23BB as a drug candidate could offer renal protective effect against rhabdomyolysis-induced AKI. In the present study, we investigated the effect of 23BB in a murine model of glycerol (GL) injection-induced rhabdomyolysis. Following GL injection, the mice developed severe AKI as indicated by acute renal dysfunction and histologic changes, accompanied by increased HDAC6 expression in the cytoplasm of tubular epithelial cells. Pharmacological inhibition of HDAC6 by 23BB pretreatment significantly reduced serum creatinine and serum blood urea nitrogen (BUN) levels as well as attenuated renal tubular damage in GL-injured kidneys. HDAC6 inhibition also resulted in reduced TdT-mediated dUTP nick-end labeling (TUNEL)-positive tubular cells, suppressed BAX, BAK, cleaved caspase-3 levels, and preserved Bcl-2 expression, indicating that 23BB exerted potent renoprotective effects by the regulation of tubular cell apoptosis. Moreover, GL-induced kidney injury triggered multiple signal mediators of endoplasmic reticulum (ER) stress including GRP78, CHOP, IRE1α, p-eIF2α, ATF4, XBP1, p-JNK, and caspase-12. Oral administration of 23BB improved above-mentioned responses in injured kidney tissues and suggested that 23BB modulated tubular cell apoptosis via the inactivation of ER stress. Overall, these data highlighted that renal protection of novel HDAC6 inhibitor 23BB is substantiated by the reduction of ER stress-mediated apoptosis in tubular epithelial cells of rhabdomyolysis-induced AKI.

## Introduction

Acute kidney injury (AKI), characterized by a rapid decline of the glomerular filtration rate, is a serious clinical problem correlated with an aggressive disease course, high rates of mortality and increased risk of chronic kidney diseases (CKD) (Venkatachalam et al., 2015). Rhabdomyolysis, accounts for 15% of AKI cases (Chatzizisis et al., 2008), could be induced by different conditions including metabolism disorders, trauma, drugs, and toxins (e.g., statins, alcohol, and cocaine), infections, etc. (Sever et al., 2006; Bosch et al., 2009).

Although the detailed mechanisms have not been fully comprehended, it has been well-established that endoplasmic reticulum (ER) stress-mediated apoptosis of tubular epithelium cells played crucial roles in rhabdomyolysis-induced AKI (Feng et al., 2016). Organelle-mediated stress, particularly ER stress, has recently emerged as a major pathophysiological paradigm underlying apoptosis. The presence of misfolded proteins and other stresses lead to the activation of an adaptive program by the ER, known as the unfolded protein response (UPR), to restore protein-folding homeostasis (Walter and Ron, 2011). Initiation of the canonical UPR engages three distinct signaling branches, which are mediated by pancreatic ER kinase (PERK), activating transcription factor-6 (ATF6) and inositol-requiring transmembrane kinase/endonuclease-1 (IRE-1) (Gardner and Walter, 2011; Wang and Kaufman, 2016). The UPR is also linked to the activation of stress kinases such as the c-Jun N-terminal kinase (JNK) and splicing of X-box binding protein 1 (XBP1) (Calfon et al., 2002; Kim et al., 2006). The combined action of these pathways results in the inhibition of protein translation, stimulation of protein degradation and production of chaperone proteins, triggering either recovery of ER function or cell death (Kim et al., 2006).

Many cellular functions, including apoptosis, are regulated by the acetylation of histone and non-histone proteins (Jazirehi, 2010; Liu and Zhuang, 2015). An acetyl group can be added to a lysine residue by histone acetyl transferases (HATs) or be removed by histone deacetylases (HDACs). HDACs are classified into four classes based on the structure and homology: class I HDACs (HDAC1, 2, 3, and 8); class II HDACs (HDAC4, 5, 6, 7, 9, and 10); class III HDACs (SIRT1–7); and class IV (HDAC11) (Ropero and Esteller, 2007). Among the 11 isoforms of HDACs, HDAC6 activation has been reported to be involved in the pathogenesis of rhabdomyolysis-induced AKI for the contribution to renal tubular cell apoptosis, inflammatory response, macrophage infiltration and oxidative stress (Liu and Zhuang, 2015; Shi et al., 2017). Therefore, selective inhibition of HDAC6 activity may be a promising strategy for the treatment of AKI.

**FIGURE 1:**
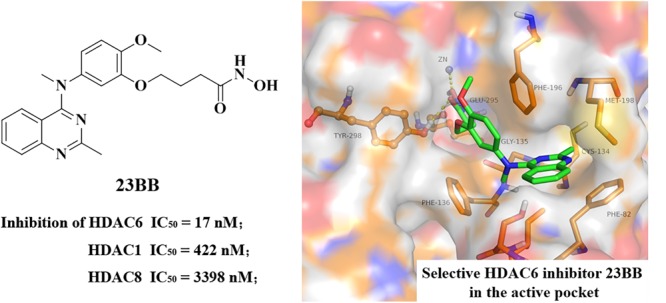
Chemical structure of 23BB and homology model of 23BB docked into HDAC6.

In our previous study, highly selective HDAC6 inhibitor 23BB has been designed, synthesized and evaluated for anti-tumor activity and safety in both solid and hematologic tumor models (**Figure 1**) (Yang et al., 2016). It demonstrated low nanomolar anti-proliferative effects against panel of cancer cell lines and more effectively inhibited the tumor growth than SAHA even at a fourfold reduced dose or ACY-1215 at the same dose. However, the therapeutic potential of 23BB as a drug candidate in rhabdomyolysis-induced AKI had not been explored. In the study, we aimed to investigate whether 23BB protected against rhabdomyolysis-induced AKI by selectively inhibiting HDAC6 and to determine the involved mechanisms.

## Materials and Methods

### Animals

This study adheres to the “Principles of Laboratory Animal Care” (National Institutes of Health publication 85-23, revised 1985) that seeks to minimize both the number of animals used and any suffering that they might experience, and is conducted in accordance with a protocol approved by Animal Care and Use Ethics Committee of Sichuan University in China (IACUC number: 2017080A). Twenty-four healthy 8-week male C57BL/6 mice were housed in a controlled environment (constant temperature at 20 ± 2°C and humidity at 50–60% with a 12-h light and 12-h dark cycle) and had free access to standard laboratory food and water. The mice were housed for 1 week of adaptation before further research.

### Glycerol-Induced AKI Model and 23BB Administration

The 24 mice were randomly divided into three groups (*n* = 8 per group): the control group, the glycerol group, and the 23BB group. The mice in the glycerol group were injected with 50% glycerol dissolved in 0.9% normal saline (10 μL/g body weight) at bilateral back limbs to elicits rhabdomyolysis-induced AKI model. The mice in the control group received an intramuscular injection of the same volume of vehicle (saline). As for the 23BB group, 23BB was dissolved in PEG and diluted in 0.9% normal saline to be orally administered at a dose of 40 mg/kg/d for 3 days before the glycerol injection.

The mice were sacrificed at 24 h after the glycerol injection. Blood sample was collected and stored at -80°C. The upper half of the left kidney was quickly removed and fixed in 10% phosphate buffered formalin for PAS staining, IHC and TUNEL assay. The lower half of the left kidney was fixed in 2.5% glutaraldehyde for 2 h at 4°C and processed for transmission electron microscope. The right kidney was quickly removed and frozen in liquid nitrogen, then stored at -80°C until processed for immunoblot analysis and immunofluorescence staining.

### Serum Analysis

Serum creatinine (sCr), blood urea nitrogen (BUN) and serum creatine kinase (CK) levels were evaluated by high performance liquid chromatography (HPLC) conducted by the Institute of Drug Clinical Trial and the GCP center of West China Hospital. The AKI model was considered established when the level of serum creatinine of the treatment group rose up to 2 times of their control littermates.

### Histologic Examination

The upper half of the left kidney, fixed in 10% phosphate buffered formalin, was dehydrated in a graded series of alcohol concentrations and embedded in paraffin. Kidney blocks were cut into 2 μm sections and then subject to PAS staining for morphologic analysis and TUNEL staining for cell apoptosis.

Periodic acid-Schiff stained tissue sections were viewed by light microscopy at magnifications of ×200 or ×400. For semi-quantitative analysis of morphological changes, two sections were randomly selected from each sample of at least 3 for every group and 10 fields were randomly selected at a magnification of ×200 from each section in periodic acid-Schiff staining. Histopathological changes were evaluated by the percentage of injured/damaged renal tubules, as indicated by tubular lysis, dilation, disruption, and cast formation. Tissue damages were scored on a scale of 0–4, with 0, 1, 2, 3, and 4 corresponding to 0%, <25%, 26%–50%, 51%–75%, ≥76% of injured/damaged renal tubules, respectively.

TUNEL stained tissue sections were viewed by light microscopy at magnifications of ×200 or ×400. The sections were stained according to the manufacturer’s protocol (Roche Molecular System, Branchburg, NJ, United States). Positive cells were counted at magnification of ×200, and at least 10 fields per section for each sample were examined (*n* = 8).

### Electron Microscopy

After being fixed in cold 2.5% glutaraldehyde for 2 h at 4°C, kidney tissues were washed with phosphate-buffered saline (PBS) (0.2 mol/L, pH 7.4) for 2 h, fixed with 1% osmic acid for 2 h, and then washed six times with PBS for 10 min per wash. The samples were dehydrated with ethanol and cleaned with epoxypropane. They were embedded in EPON 812 overnight at room temperature. Ultrathin sections (40–60 nm) were cut (EM UC61rt, Leica) and stained with uranyl acetate/lead citrate. These sections were subsequently visualized using a transmission electron microscope (H-7650, Hitachi).

### Immunoblot Analysis

Mouse kidney cortexes were dissected and homogenized in radio immune precipitation (RIPA) lysis buffer (P0013B, Beyotime Biotechnology, China). After centrifugation at 13,000 rpm for 15 min at 4°C, the supernatant was collected, and protein concentrations were determined using a bicinchoninic acid (BCA) Protein Assay Kit (Beyotime Institute of Biotechnology). Bovine serum albumin was used as the standard. Equal amounts of protein lysate were loaded directly on 10–12% SDS-PAGE, transferred onto polyvinylidene difluoride (PVDF) Membrane for Protein Blotting(0.2 μm, Bio-Rad Laboratories, Inc.). The membranes were blocked with 5% non-fat dry milk (w/v) in Tris-buffered saline with 0.1% Tween-20 (TBS-T) for 1 h at room temperature and then incubated with indicated primary antibodies overnight at 4°C. After being rinsed thrice with TBS-T at 5-min intervals, the membranes were incubated with horseradish peroxidase-labeled goat anti-rabbit IgG (1:2000 dilution; Biosynthesis Biotechnology Co., Ltd., Beijing, China) or goat anti-mouse IgG (1:2000 dilution; Biosynthesis Biotechnology Co., Ltd., Beijing, China) for 1 h. Immunoblots were visualized using the Immobilon Western Chemiluminescent HRP Substrate (Millipore Corporation, Billerica, MA, United States) with Bio-Rad Chemi Doc MP. All immunoblot analysis data are from experiments performed in triplicate. Densitometry analysis was performed using ImageJ6.0 software (National Institutes of Health, Bethesda, MD, United States).

### Immunofluorescence Staining

Renal tissues were fixed in 4.5% buffered formalin, dehydrated, and embedded in paraffin. For immunofluorescent staining, the tissue sections were rehydrated and blocked with PBS+5% normal goat serum for 1 h, after which they were labeled with indicated antibodies in a humidified chamber overnight at -4°C. The sections were exposed to Cy5 red-labeled or FITC green-labeled secondary antibodies (Jackson ImmunoResearch Inc., West Grove, PA, United States). The nuclei were counterstained with DAPI (1:500, Life Technologies Corporation, OR, United States). the images were captured with AxioCam HRc digital camera (Carl Zeiss).

### Immunohistochemistry

After fixation in 10% phosphate buffered formalin overnight, the fixed kidneys were dehydrated through a graded series of ethanol, embedded in paraffin, sectioned (5 μm), and mounted on glass slides. The slides were blocked with 2.5% normal goat serum and incubated with primary antibodies at 4°C. The slides were washed thrice in PBS, and VECTASTAIN ABC Kit (Vector, Burlingame, CA, United States) was used for staining following the manufacturer’s instruction. The sections were counterstained with hematoxylin. Images were captured using an AxioCam HRc digital camera (Carl Zeiss).

### Primary Antibodies

Anti-HDAC6 (sc11420, Santa Cruz), anti- acetyl-histone H3 (9649, Cell Signaling Technology), anti-BAX (ab32503, Abcam), anti-Bcl-2 (ab3214, Abcam), anti-cleaved caspase-3 (9664, Cell Signaling Technology), anti-phospho-JNK (4668, Cell Signaling Technology), anti-JNK (ab208035, Abcam), anti-phospho-eIF2α (3398, Cell Signaling Technology), anti-eIF2α (5324, Cell Signaling Technology),anti-phospho-PERK (sc32577, Santa Cruz) anti- GRP78 (ab108613, Abcam), anti- IRE1α (ab48187, Abcam), anti-CHOP (2895, Cell Signaling Technology), anti-ATF4 (11815, Cell Signaling Technology), anti- XBP1 (ab37152, Abcam), anti-caspase-12 (ab62484, Abcam).

### Statistical Analysis

All experiments were performed in triplicate unless otherwise stated. Data are presented as mean ± SEM. Data were subjected to analysis of variance (ANOVA) followed by Tukey’s *post hoc* test, and unpaired Student’s *t*-test for comparison between two groups. *P*-values < 0.05 were considered statistically significant.

## Results

### Novel HDAC6 Inhibitor 23BB Alleviated Rhabdomyolysis-Induced AKI

**FIGURE 2:**
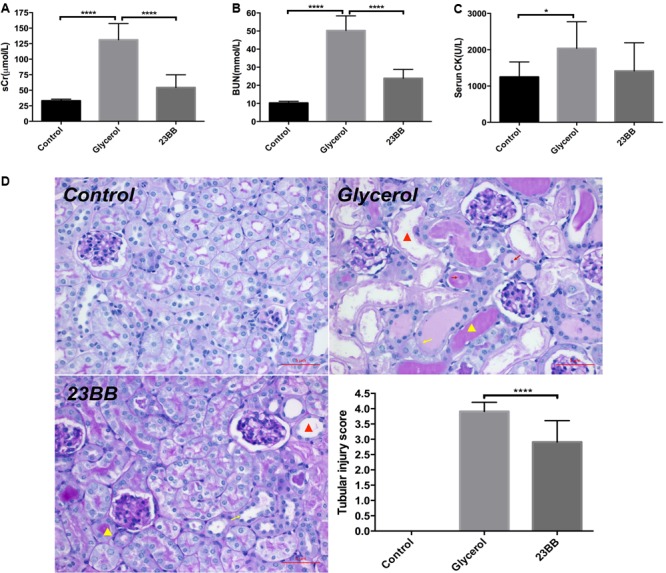
Inhibition of HDAC6 with 23BB alleviates glycerol-induced AKI. **(A)** Serum creatinine (sCr). **(B)** Serum blood urea nitrogen (BUN). **(C)** Serum creatine kinase (CK). **(D)** Photomicrographs (X400) illustrate periodic acid-Schiff (PAS) staining of the kidney tissues (red triangle: tubular dilatation; red arrow: necrosis; yellow triangle: cast formation; yellow arrow: loss of brush border). Histologic changes were scored based on the scale described in the Section “Materials and Methods.” Data are represented as the means ± SE (*n* = 6 for each group). ^∗∗∗∗^*P* < 0.0001; ^∗^*P* < 0.05.

To determine whether 23BB may have a renal protective effect by targeting HDAC6, we examined the renal function and pathological changes in a mouse model of rhabdomyolysis-induced AKI. As demonstrated in **Figure 2**, serum creatinine (sCr), blood urea nitrogen (BUN) and serum creatine kinase (CK) were markedly elevated at 24 h after GL injection. Pretreatment of 23BB at a dose of 40 mg/kg/d for 3 d significantly improved acute renal dysfunction without influencing the level of serum CK. Consistent with improved kidney function by 23BB administration, a PAS-stained section showed less tubular dilatation, swelling, necrosis, cast formation and preservation of a brush border in the GL+23BB administered group as compared to that of GL group, which was indicated by the quantification of the kidney injury score (**Figure 2D**).

Taken together, these data suggested that compound 23BB protected against rhabdomyolysis-induced AKI via the inhibition of HDAC6 activity.

### HDAC6 Inhibitor 23BB Enhanced the Acetylation of Histone H3 in the Kidney of Rhabdomyolysis-Induced AKI

**FIGURE 3:**
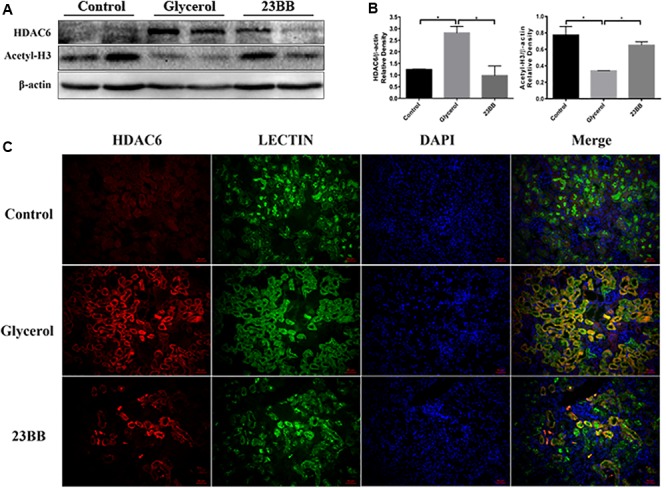
23BB inhibits the expression of HDAC6 and enhances the acetylation of histone H3. **(A)** The kidney tissue lysates were subjected to immunoblot analysis with indicated antibodies against HDAC6 and acetylated histone H3. **(B)** Expressions of HDAC6 and acetylated histone H3 were quantified by densitometry and normalized with β-actin. Data are represented as the means ± SE (*n* = 6 for each group). ^∗^*P* < 0.05. **(C)** Immunofluorescence staining of HDAC6 and Lectin in the kidney tissue sections. Lectin was used as a marker of tubular epithelial cells.

Inhibition of HDAC can be reflected by the increased expression of acetyl histone H3 (Shi et al., 2017). To understand the inhibitory effect of 23BB on HDAC6 activity, we examined the expression of acetyl histone H3 and HDAC6 by immunoblot analysis. As exhibited in **Figures 3A,B**, the increased expression of HDAC6 was detected in GL-injected group, which was suppressed by 23BB pretreatment. Meanwhile, GL group showed a remarkable decrease of acetyl histone H3 level compared to that of control group, and the change was restored by 23BB administration.

Furtherly, to investigate whether HDAC6 was expressed in renal tubular epithelia cells, kidney tissues were stained by HDAC6 and Lotus tetragonolobus lectin (Lectin), a proximal epithelial cell marker. As demonstrated in **Figure 3C**, we found that HDAC6 was minimally expressed in the control group, but remarkably upregulated in GL group and was co-stained with Lectin. Oral administration of 23BB significantly suppressed the HDAC6 expression, which was consistent with results of immunoblot analysis. These findings indicated that glycerol induced the upregulation of HDAC6 in tubular epithelial cells, and that 23BB largely diminished this response.

### Inhibition of HDAC6 Activity Decreased Renal Tubular Cell Apoptosis in Rhabdomyolysis-Induced AKI

**FIGURE 4:**
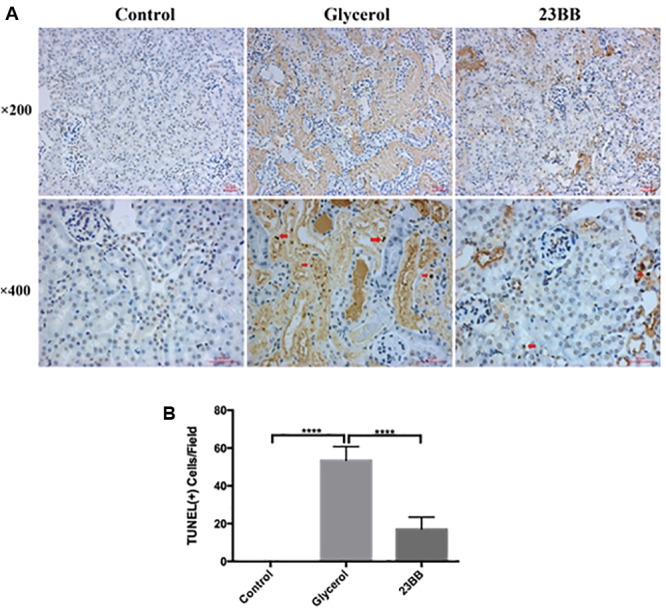
Inhibition of HDAC6 activity decreased renal tubular cell apoptosis. **(A)** TUNEL staining of kidney tissue sections were collected at ×200 and ×400 magnifications (red arrow: TUNEL positive cells). **(B)** Positive TUNEL staining cells were counted and expressed as means ± SE. ^∗∗∗∗^*P* < 0.0001.

Renal tubular cell apoptosis was a prominent feature in the development of rhabdomyolysis-induced AKI (Tang et al., 2013). To examine the role of HDAC6 in the model, we first analyzed the tubular cell apoptosis by TdT-mediated dUTP nick-end labeling (TUNEL) staining. As expected, large number of TUNEL-positive renal tubular cells were observed in the injured kidney after GL injection. In comparison, 23BB administration diminished TUNEL-positive tubular cells (**Figure 4**).

**FIGURE 5:**
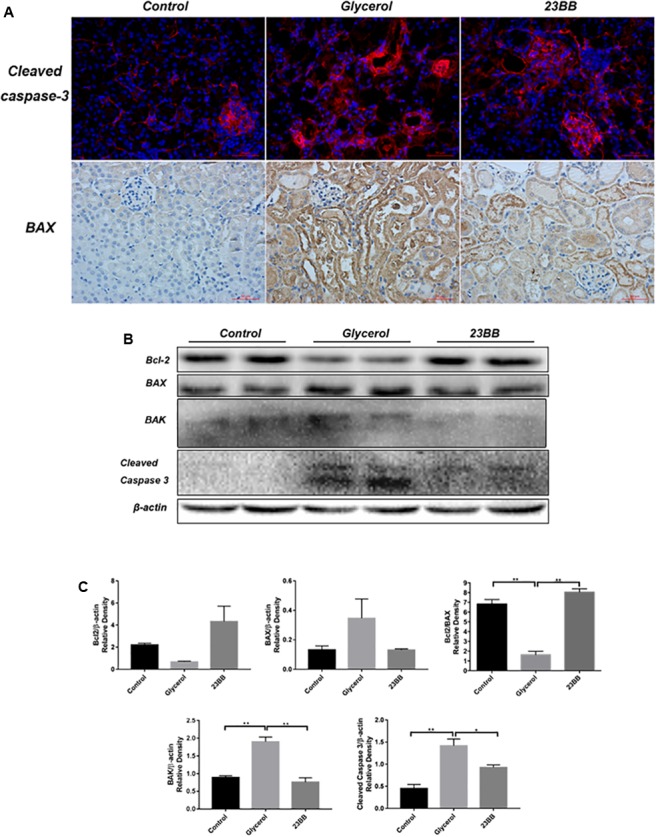
23BB inhibits the expression of apoptosis related proteins. **(A)** Immunofluorescence staining was performed to detect expression of cleaved caspase-3 in kidney tissue sections in each group, immunohistochemistry illustrated BAX-stained kidney sections in each group. **(B)** The kidney tissue lysates were subjected to immunoblot analysis with indicated antibodies against BAX, Bcl-2, BAK and cleaved caspase-3. **(C)** Expressions of BAX, Bcl-2, BAK and cleaved caspase-3 were quantified by densitometry and normalized with β-actin. Data are represented as the means ± SE (*n* = 6 for each group). ^∗∗^*P* < 0.01; ^∗^*P* < 0.05.

Caspase-3 was the key executioner which ultimately modified proteins responsible for cell apoptosis (Savitskaya and Onishchenko, 2015). B-cell lymphoma 2 (Bcl-2) family consisted of a group of essential regulatory factors in cell apoptosis. Bcl-2 was a representative group I member which was anti-apoptotic, while Bcl-2-associated X (BAX) and Bcl-2 homologous antagonist/killer (BAK) were representative group II members which were apoptotic (Cory and Adams, 2002; Youle and Strasser, 2008). In the study, we further examined the expression of BAX, BAK, Bcl-2 and cleaved caspase-3 in the kidney after GL injection with or without 23BB administration by immunoblot analysis (**Figure 5**). Consistent with TUNEL staining results, GL injection induced tubular cell apoptosis, as assessed by the up-regulation of BAX, BAK and down-regulation of Bcl-2, besides boosting cleaved caspase-3 levels. However, 23BB administration led to marked renal protection against GL-induced apoptosis, as judged by restored the expressions of apoptosis-related markers. Immunofluorescence staining or IHC of the proteins were also conducted and the expressions in renal tubular cells were consistent with the results of immunoblot analysis.

Collectively, these data demonstrated that the selective inhibitory effect of 23BB on HDAC6 activity contributed to ameliorated renal tubular cell apoptosis in rhabdomyolysis-induced AKI.

### Pharmacological HDAC6 Inhibition Suppressed ER Stress and UPR in Rhabdomyolysis-Induced AKI

**FIGURE 6:**
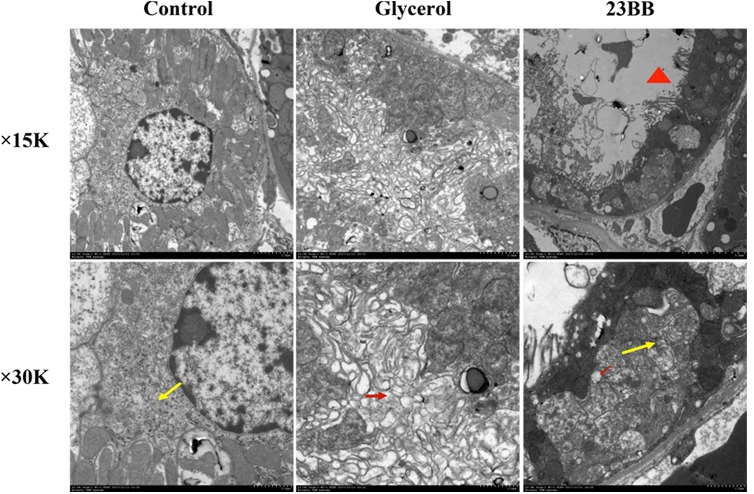
23BB administration attenuates endoplasmic reticulum expansion. Photomicrographs (×15k and ×30k) collected by transmission electron microscope (yellow arrow: normal endoplasmic reticulum; red arrow: expansion of endoplasmic reticulum; red triangle: loss of brush border).

By transmission electron microscopy (**Figure 6**), in the GL group, we observed large amount of swelling ER in the cytoplasm of renal tubular cells, which was not detected in the control group. Other ultrastructural changes including mitochondrial swelling and loss of brush border were also observed in GL group (Supplementary Figure 1). The administration of 23BB dramatically reduced swelling ER, while the mitochondrial swelling and the brush border loss were still detected. We hypothesized that 23BB ameliorated apoptosis through the suppression of ER stress.

**FIGURE 7:**
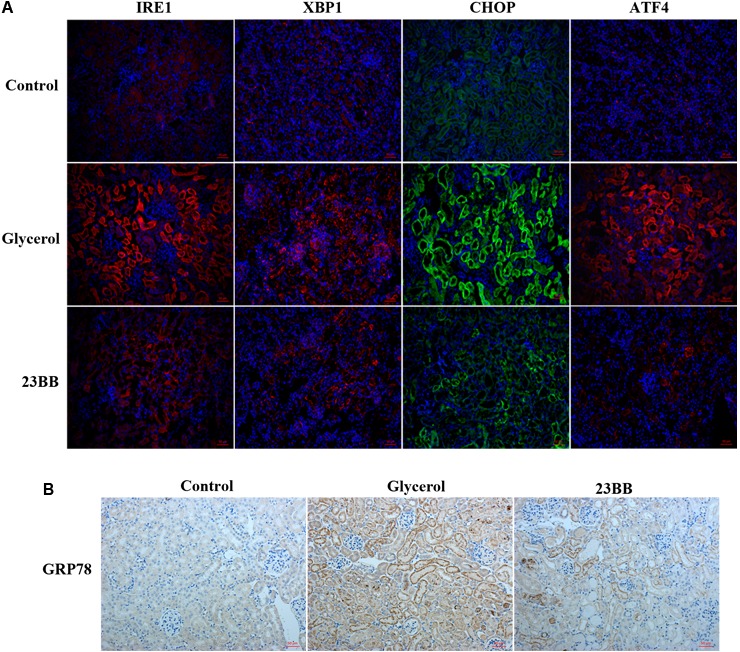
Inhibition of HDAC6 activity down-regulated ER stress related proteins in renal tubular cells. **(A)** Immunofluorescence staining was performed to detect expression of IRE1, XBP1, CHOP, and ATF4 in kidney tissue sections in each group. **(B)** Immunohistochemistry illustrated GRP78-stained kidney sections in each group.

**FIGURE 8:**
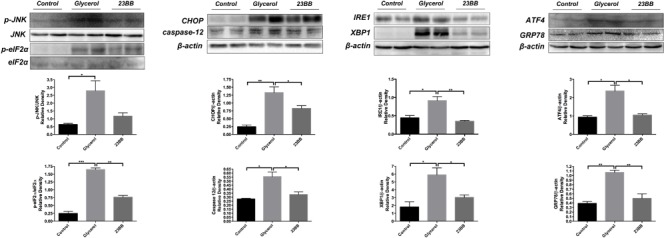
23BB administration suppressed expression level of ER stress proteins. The expression of GRP78, IRE1α, p-eIF2α, ATF4, CHOP, p-JNK, caspase-12, and XBP1, as measured by immunoblot analysis, in kidney tissue sections in each group. The densitometry values of p-JNK were normalized with JNK. The densitometry values of p-eIF2α were normalized with eIF2α. The densitometry values of other proteins were normalized with β-actin. Data are represented as the means ± SE (*n* = 6 for each group). ^∗^*P* < 0.05.

To validate the hypothesis, we first conducted immunoblot analysis of ER stress related proteins including GRP78, IRE1α, p-eIF2α, ATF4, CHOP, p-JNK, caspase-12, and XBP1. As indicated in **Figures 7, 8**, GL injection induced the upregulation of ER stress related proteins in the injured kidney, which was significantly suppressed by 23BB administration. Furthermore, we performed IHC staining of GRP78 and immunofluorescence staining of IRE1α, ATF4, CHOP and XBP1. We found these proteins were minimally expressed in the control group, but remarkably upregulated in glycerol group, predominantly located in the renal tubules. Pretreatment of HDAC6 inhibitor 23BB significantly suppressed ER stress related proteins. Thus, these findings highlighted that 23BB administration inhibited the activation of ER stress in glycerol-induced AKI.

## Discussion

**FIGURE 9:**
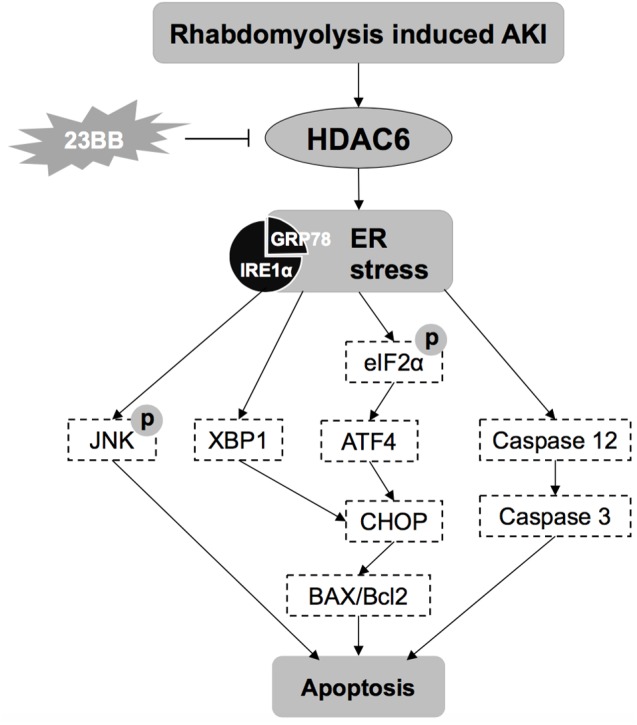
The involved mechanisms of 23BB against rhabdomyolysis-induced AKI.

Previous studies have confirmed that HDAC6 contributed to the pathogenesis of rhabdomyolysis-induced AKI and selective inhibition might be a promising strategy for the treatment of AKI (Shi et al., 2017). Compound 23BB as a highly selective and potent HDAC6 inhibitor has been designed, synthesized and evaluated for anti-tumor activity in our lab (Yang et al., 2016). While in the study, we found that pharmacological inhibition of HDAC6 by 23BB improved acute renal dysfunction indicated by reduced sCr and BUN levels in a GL-induced AKI model. Pretreatment of 23BB alleviated renal tubular damage and attenuated ER stress-mediated apoptosis in the tubular epithelial cells of GL-injured kidney tissues. The involved mechanism of 23BB against rhabdomyolysis-induced AKI has been summarized in **Figure 9**. Our data demonstrated the protective effects of 23BB on rhabdomyolysis-induced AKI and provided new evidence regarding the potential therapeutic effects on AKI.

Apoptotic cell death is usually a response to cell microenvironment (Dang et al., 2015; Su et al., 2015). Apoptosis requires the activation of lethal molecules and the inactivation of prosurvival ones (Youle and Strasser, 2008). Apoptotic pathways are active in the tubular epithelium induced by caspase cascade activation, mitochondrial injury, and ER stress, etc. (Savitskaya and Onishchenko, 2015). Apoptosis also promotes renal epithelial cell loss that characterizes acute kidney diseases (Havasi and Borkan, 2011; Linkermann et al., 2014). Accumulating evidence has demonstrated that the caspase-3 activation is the predominant mechanism responsible for the apoptosis of renal tubular cells in rhabdomyolysis-induced AKI (Linkermann et al., 2014; Feng et al., 2016). In our study, HDAC6 inhibition by 23BB blocked the cleavage of caspase-3 in the injured kidney, suggesting that HDAC6 mediated the activation of caspase-3. Another crucial regulatory factor in apoptosis is the Bcl-2 family which could be divided into two major classes based on their functions and structures: group I proteins that are anti-apoptotic and group II proteins that are apoptotic (Cory and Adams, 2002; Youle and Strasser, 2008). Thus, the inhibition of group I proteins and/or the activation of group II proteins can successfully induce cell apoptosis. Herein, we evaluated the impact of 23BB on the level of anti-apoptotic Bcl-2 and apoptotic BAX/BAK. We found that the blockage of HDAC6 by 23BB significantly suppressed BAX/BAK and preserved Bcl-2 in the kidney tissues of rhabdomyolysis-induced AKI. Therefore, 23BB possessed inhibitory effects on tubular epithelial cell apoptosis in the pathogenesis of rhabdomyolysis-induced AKI.

Endoplasmic reticulum stress could be triggered by different stimulatory signals in AKI, including mutant protein aggregation, hypoxia, energy deprivation, and metabolic dysfunction (Taniguchi and Yoshida, 2015). Declined protein-folding capacity of ER leads to the accumulation of misfolded proteins, initiating ER stress. Overwhelming ER stress induces tubular cell apoptosis via three typical signal pathways, PERK-eIF2-ATF4, IRE1-XBP1 and ATF6 pathway (Gardner and Walter, 2011). Under non-stressed conditions, glucose-regulated protein (GRP78) binds to its client proteins PERK, ATF6, and IRE1, preventing their signaling. However, when ER is overloaded, GRP78 binds to the unfolded proteins in the ER, freeing its client proteins, which then serves as the primary mediators of UPR signaling (Walter and Ron, 2011; Wang and Kaufman, 2016). UPR is a cellular quality control mechanism with two primary functions: first, to promote survival during ER stress by chaperoning proteins for re-folding and halting transcription and translation until homeostasis is restored; second, to signal CCAAT/enhancer-binding protein-homologous protein (CHOP) mediated apoptosis when homeostasis could not be re-established (Gorman et al., 2012). During UPR, PERK is released from its chaperone protein GRP78 to permit phosphorylation of eIF2α, leading to the activation of ATF4 and CHOP. Released IRE1 targets the downstream XBP1 and JNK, resulting in cell apoptosis (Calfon et al., 2002; Gardner and Walter, 2011). Apart from that, caspase-12, which is only expressed in rodents, is another major mediator of ER stress-mediated apoptosis that responds to ER stress and induces the caspase-3 cleavage to initiate apoptosis (Morishima et al., 2002).

Considerable evidence suggested some connection between HDAC inhibitors and ER stress in other disease models including lymphoma (Cosenza et al., 2017), breast cancer (Komatsu et al., 2013) and Duchenne muscular dystrophy (Consalvi et al., 2014). Previous study has reported that class I HDACs could localize ER, bind to GRP78, and selectively activate the UPR (Kahali et al., 2012). HDAC6, which primarily resides in the cytoplasm, contributed to the acetylation of ER-localized chaperone protein GRP78 (Li et al., 2016). In our study, we found that the inhibition of HDAC6 by 23BB significantly suppressed ER stress, as evidenced by the decreased expression of GRP78. The downstream UPR was also alleviated, as indicated by the diminished expression of IRE1α, XBP1, p-eIF2α, ATF4. Moreover, 23BB pretreatment down-regulated the expression of mediators of ER stress-mediated apoptosis, including CHOP, p-JNK and caspase-12. In a nutshell, the protective effect of 23BB against tubular epithelial cell apoptosis is mediated via the suppression of ER stress.

Notably, the functional significance of HDACs in rhabdomyolysis-induced AKI remains controversial (Hui and Chiang, 2014). Given the fact that our study showed that HDAC6 inhibition by selective small molecule offered a protective effect on the injured kidney in the pathological condition, there were reports of blocking class I HDACs by MS-275 resulted in worsening renal dysfunction and enhancing cell apoptosis with caspase-3 activation in the same AKI model (Tang et al., 2014). In support of our observations, administration of TA, another HDAC6 selective inhibitor, was reported to exert a similar protection in rhabdomyolysis-induced AKI model (Shi et al., 2017). This debate also applies to other AKI models. In cisplatin-induced AKI models, on one hand, there are studies showing that TSA suppresses cisplatin-induced tubular epithelial cell apoptosis through the suppression of p53 and restoration of CREB-mediated transcription (Arany et al., 2008; Dong et al., 2010). On the other hand, the pro-apoptotic effect of HDAC inhibitor SAHA and TSA were also reported in tubular epithelial cell apoptosis (Dong et al., 2008).

A possible explanation is that different classes of HDACs may serve distinct roles in activating various signaling pathways that lead to the development of AKI. To what extent each isoform of HDAC contributes to AKI is not fully comprehended. Thus, these non-selective or partially selective HDAC inhibitors usually lead to undesirable biological responses that accelerate pathologic changes. HDAC6, which is primarily expressed in the cytoplasm, removes the acetyl group from lysine residues in many non-histone substrates. In contrast to the lethal effect of HDAC1-3 genetic ablation, HDAC6-knocked out mice are viable and fertile (Ran et al., 2016). These results supported that HDAC6 selective inhibitor 23BB is a safe agent with fewer side effects than pan-HDAC inhibitors and may be a promising agent for the treatment of AKI.

## Conclusion

In summary, our findings demonstrated that highly selective HDAC6 inhibitor 23BB protects against rhabdomyolysis-induced AKI via the regulation of ER stress-mediated apoptosis in tubular epithelial cells. Thus, compound 23BB as a promising drug candidate may be a novel strategy for the treatment of AKI.

## Author Contributions

LM and PF conceived and designed the experiments. YuF, RH, FG, YL, JX, SL, MS, and YaF performed the experiments. YuF, LL, JL, and RH analyzed the data. YuF and LM wrote the paper. YuF, RH, FG, YL, JX, SL, MS, LL, JL, YaF, LM, and PF approved the final version of the manuscript.

## Conflict of Interest Statement

The authors declare that the research was conducted in the absence of any commercial or financial relationships that could be construed as a potential conflict of interest.
