# Supplementary material for: Selective Histone Deacetylase 6 Inhibitor 23BB Alleviated Rhabdomyolysis-Induced Acute Kidney Injury by Regulating Endoplasmic Reticulum Stress and Apoptosis
Source: Front Pharmacol. 2018 Mar 26;9:274. doi: 10.3389/fphar.2018.00274 (PMC5879111; doi:10.3389/fphar.2018.00274)
Supplement: Supplementary file 1 [file Data_Sheet_1.docx]

Supplementary Material

Selective Histone Deacetylases 6 Inhibitor 23BB Alleviated Rhabdomyolysis-Induced Acute Kidney Injury by Regulating Endoplasmic Reticulum Stress and Apoptosis

**Yuying Feng ^1a^, Rongshuang Huang ^1a^, Fan Guo ^2^, Yan Liang ^2^, Jin Xiang ^3^, Song Lei ^4^, Min Shi ^1^, Lingzhi Li ^1^, Jing Liu ^1^, Yanhuan Feng ^1^, Liang Ma ^1^*, and Ping Fu ^1^***

^1^ Kidney Research Institute, Division of Nephrology, West China Hospital of Sichuan University, Chengdu, China.

^2^ Core Facility of West China Hospital, West China Hospital of Sichuan University, Chengdu, China.

^3^Laboratory of Clinical Pharmacology, West China Hospital of Sichuan University, Chengdu, China.

^4^ Department of Pathology, West China Hospital of Sichuan University, Chengdu, China.

^a^ F. Feng and R. Huang contributed equally to the work.

*** Correspondence:**L. Ma, and P. Fu
liang_m@scu.edu.cn and fupinghx@163.com

## Supplementary Figures


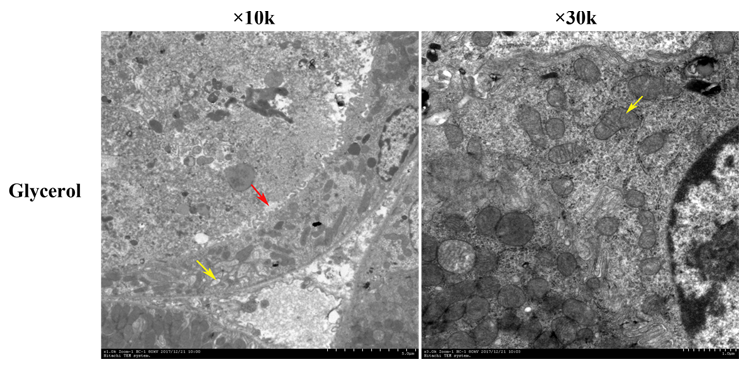


**Supplementary Figure 1.** Photomicrographs (×10k and ×30k) collected by transmission electron microscope (yellow arrow: mitochondrial swelling; red arrow: loss of brush border)
